# Evaluation of nutrient and energy sources of the deepest known serpentinite-hosted ecosystem using stable carbon, nitrogen, and sulfur isotopes

**DOI:** 10.1371/journal.pone.0199000

**Published:** 2018-06-15

**Authors:** Yuji Onishi, Toshiro Yamanaka, Tomoyo Okumura, Shinsuke Kawagucci, Hiromi Kayama Watanabe, Yasuhiko Ohara

**Affiliations:** 1 Graduate school of Natural Science and Technology, Okayama University, Kita-ku, Okayama, Okayama, Japan; 2 Department of Subsurface Geobiological Analysis and Research (D-SUGAR), Japan Agency for Marine-Earth Science and Technology, Yokosuka, Kanagawa, Japan; 3 Department of Marine Biodiversity Research, Japan Agency for Marine-Earth Science and Technology, Yokosuka, Kanagawa, Japan; 4 Hydrographic and Oceanographic Department of Japan, Kasumigaseki, Chiyoda-ku, Tokyo, Japan; Museum National d'Histoire Naturelle, FRANCE

## Abstract

The Shinkai Seep Field (SSF) in the southern Mariana forearc discovered in 2010 is the deepest (~5,700 m in depth) known serpentinite-hosted ecosystem dominated by a vesicomyid clam, *Calyptogena* (*Abyssogena*) *mariana*. The pioneering study presumed that the animal communities are primary sustained by reducing fluid originated from the serpentinization of mantle peridotite. For understanding the nutrient and energy sources for the SSF community, this study conducted four expeditions to the SSF and collected additional animal samples such as polychaetes and crustaceans as well as sediments, fragments of chimneys developing on fissures of serpentinized peridotite, seeping fluid on the chimneys, and pore water within the chimneys. Geochemical analyses of seeping fluids on the chimneys and pore water of the chimneys revealed significantly high pH (~10) that suggest subseafloor serpentinization controlling fluid chemistry. Stable isotope systematics (carbon, nitrogen, and sulfur) among animals, inorganic molecules, and environmental organic matter suggest that the SSF animal community mostly relies on the chemosynthetic production while some organisms appear to partly benefit from photosynthetic production despite the great depth of SSF.

## Introduction

Serpentinization of ultramafic rocks such as peridotite produces H_2_-rich fluid [1] that can yield CH_4_ by CO_2_ reduction through biotic (e.g., microbial methanogenesis) and/or abiotic (e.g., Fischer-Tropsch Type reaction) processes [2–5]. H_2_ and CH_4_ can be oxidized exothermically, coupled with sulfate reduction by microbes such as sulfate-reducing bacteria (SRB) in the anoxic habitats, leading to H_2_S production [6]. Consequently, serpentinization-associated fluids can provide relevant energy sources for chemolithoautotrophic primary producers using H_2_ and H_2_S, as well as methanotrophs using CH_4_. Such productivity often becomes the basis for nourishing high-biomass faunal communities (e.g. [7]).

In 2010, an animal community composed mainly of vesicomyid clam, *Calyptogena* (*Abyssogena*) *mariana* [8], was discovered at *c*. 5500–5700 m depth in a site on the inner trench slope of the southern Mariana forearc named the Shinkai Seep Field (SSF) [9]. Since this clam relies on endosymbiotic chemoautotroph for nutrient, this community was presumed to be chemosynthesis-based [8, 9]. Subsequent expeditions to the SSF until 2015 revealed numerous white chimneys composed of brucite and/or calcium carbonate minerals [10]. Biological surveys in both the clam colony and the chimney sites also revealed a higher diversity of animals, including for example *Phyllochaetopterus* polychaetes and a gastropod *Provanna cingulata* [9–11], which possibly feed on chemolithoautotrophic microbes. Although not visible, the presence of chemically unstable brucite-carbonate chimneys [10] and presumably chemosynthetic symbiont-associated animals strongly suggests the existence of alkaline and reducing fluid emission (or discharge). Fluids discharging along fissures of peridotites either exposed to the seafloor or covered by thin sediments is believed to be associated with the subseafloor serpentinization in the SSF, as observed in the Lost City hydrothermal field (discharging fluid temperature ≤91°C [12, 13]) and the South Chamorro Seamount (though shimmering fluid was not observed and temperature anomalies not reported [14, 15]). There has so far been no geochemical data from the SSF, however, to confirm the fluid seepage or the energetic relationship between geological processes and the faunal community there.

Stable isotopic signatures of biophilic elements have been used to elucidate the basal foods and trophic levels for animals around hydrothermal vents and hydrocarbon seeps in the deep-sea (e.g. [16, 17]). Carbon and sulfur isotopic compositions of animal tissues are used to estimate basal foods because there are negligible enrichments of ^13^C (0 to 1‰; [18]) or depletions of ^34^S (0 to 5‰; [19]) through trophic levels [20] as well as assimilation of environmental molecules by each primary producer (e.g. [21–23]). Nitrogen isotopic composition is used to assess the trophic level of a species because an increase of +3.4‰ of δ^15^N is usually considered along with increasing a trophic level [24]. The combined use of these three isotopic signatures for each constituent of an ecosystem can therefore provide an estimation of food-web structure (e.g. [17]).

The purpose of this study is at first to geochemically characterize the SSF seeping fluids that likely support animal communities there and then to elucidate energy and nutrient sources of dominant animals in the SSF. For this purpose, we conducted sampling and chemical analyses of the relevant fluids, as well as carbon, sulfur, and nitrogen isotopic analyses of the animal soft tissues and organic matter in the sediments and the chimneys.

## Materials and methods

### Study site

**Fig 1:**
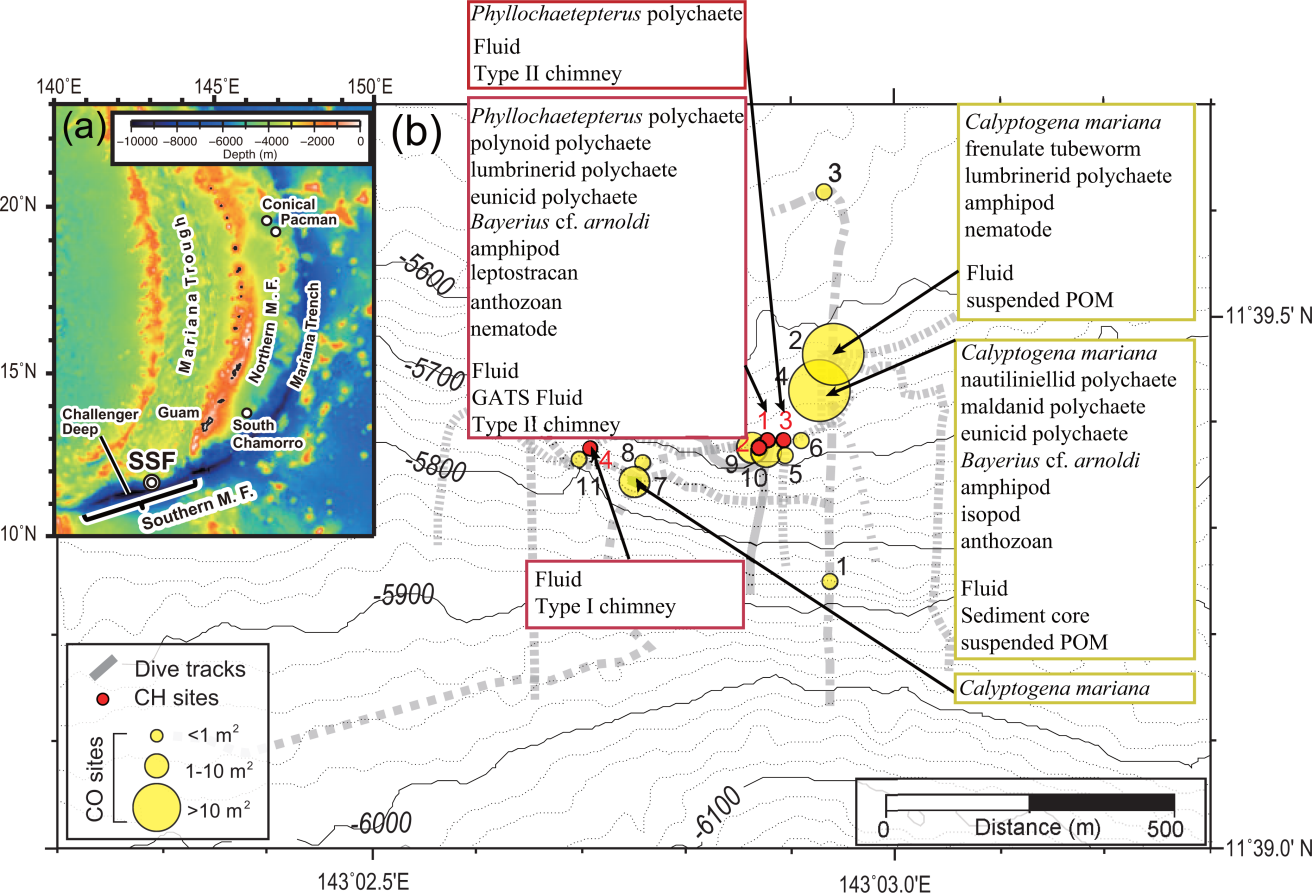
Map of the sampling sites in the SSF and detailed distribution of the CO and CH Sites. The black and red numbers beside the yellow and red circles indicate each site number (CO and CH, respectively). The size of yellow circles corresponds to the size of clam colonies (modified from ref. [10]).

The Mariana arc-trench system is a typical non accretionary convergent plate margin [25] where the Pacific Plate is subducting beneath the Philippine Sea Plate. The SSF is located on a deep inner trench slope of the southern Mariana forearc, ~80 km northeast of the Challenger Deep [9] (Fig 1A). The southern Mariana forearc is young and tectonically active, hosting serpentinized peridotite along the deep inner trench slope [9]. In the SSF, eleven clam colony sites mainly composed of *C*. *mariana* [8] (CO Sites 1–11 indicated by black numbers in Fig 1B) and four brucite-carbonate chimney structure sites (CH Sites 1–4 indicated by red numbers in Fig 1B) have been identified [10]. These chimney structures were observed to develop from the fissures of the serpentinized peridotite [10].

### Sampling procedure and on-board sample processing

#### Expeditions and dives

Animals, sediment cores, chimney fragments, and fluid samples were collected using the deep-submergence vehicle (DSV) *Shinkai 6500* during expeditions YK13-08, YK14-13, and YK15-11 of the R/V *Yokosuka*, and using the remotely operated vehicle (ROV) *Kaiko* (with vehicle *Mk-IV*) during the expedition KR16-14 of the R/V *Kairei* (Table 1 and Fig 1B). All four expeditions and sample collections were performed under the permission of the US Fish and Wildlife Service (SUP#12541–12001 for YK13-08, SUP#12541–14001 for YK14-13, SUP#12541–15002 for YK15-11, and SUP#12541–16002 for KR16-14).

**Table 1:** Expeditions and submersible dives, surveyed site locations, and samples collected.

**Expedition (SUP#)**	Dive#	Date	Site	Latitude	Longitude	Depth (m)	Collected sample (Chimney ID)
YK13-08 (12541–12001)	6K#1362	2013/9/9	CO Site 4	11°39.41'N	143°2.94'E	5629	Animal, Fluid, Sediment core, suspended POM
	6K#1365	2013/9/12	CO Site 2	11°39.44'N	143°2.95'E	5622	Animal, Fluid, suspended POM
			CH Site 1	11°39.37'N	143°2.85'E	5683−5681	Animal, Fluid, Type II chimney* (Chim 2)
	6K#1366	2013/9/13	CO Site 7	11°39.33'N	143°2.75'E	5740	Animal
YK14-13 (12541–14001)	6K#1397	2014/7/12	For Reference	11°13.58'N	139°15.18'E	4640	Fluid, suspended POM
	6K#1402	2014/7/17	CH Site 3	11°39.36'N	143°2.88'E	5689−5683	Animal, Type II chimney* (Chim 4)
	6K#1403	2014/7/18	CO Site 2	11°39.44'N	143°2.95'E	5622	Fluid, suspended POM
	6K#1404	2014/7/19	CH Site 4	11°39.28'N	143°2.93'E	5743	Type I chimne* (Chim 5 and 6)
YK15-11 (12541–15002)	6K#1432	2015/7/14	For Reference	11°40.65'N	142°59.26'E	4550	Sediment core
	6K#1433	2015/7/15	CH Site 3	11°39.36'N	143°2.88'E	5689−5683	Type II chimney *(Chim 10)
			CH Site 4	11°39.28'N	143°2.93'E	5743	Fluid, Type I chimne * (Chim 9)
			CH Site 3	11°39.36'N	143°2.88'E	5689−5683	Fluid, Type II chimney§ (Chim 18)
			CH Site 4	11°39.28'N	143°2.93'E	5743	Type I chimney§ (Chim 16)
			CH Site 3	11°39.36'N	143°2.88'E	5689−5683	GATS Fluid

*; Chimney ID listed in Okumura et al., 2016a [10]

§; in-group Chimney ID

#### Animals

Animal sampling was conducted using a suction sampler and a scoop operated by the manipulators of the DSV *Shinkai 6500* during the dives 6K#1362, 6K#1365, and 6K#1366 in expedition YK13-08 and dive 6K#1404 in expedition YK14-13. Each organism was identified on-board based on their morphology, before being frozen and preserved at −80°C in a deep-freezer for on-shore analyses.

*Calyptogena* clams were collected from three sites (CO Sites 2, 4, and 7 in Fig 1B) with a scoop, and simultaneously other animal species such as the snail *Bayerius* cf. *arnoldi*, polychaetes (unidentified species of Maldanidae, Lumbrineridae, and Eunicidae), crustaceans (unidentified species of amphipods and isopods), nematodes (species unidentified), and anthozoans (species unidentified) were collected in the CO Sites 2 and 4. *Bayerius* cf. *arnoldi* and the anthozoans were observed on the seafloor during the dive surveys, while the other animals were not seen on the seafloor due to its smaller size and therefore it is unknown whether they lived on or inside sediment. In CO Site 2, frenulate tubeworms on the sediment were collected with manipulators. Moreover, nautiliniellid polychaetes were found inside the clam shells. In CH Sites 1 and 3, animals on the chimney surface such as *B*. cf. *arnoldi*, polychaetes (unidentified species of *Phyllochaetopterus*, Polynoidae, Lumbrineridae, and Eunicidae), crustaceans (unidentified species of amphipods and leptostracans), nematodes (species unidentified), and anthozoans (species unidentified) were collected by a suction sampler.

#### POM in seawater

For suspended particulate organic matter (POM) analyses, seawater samples were collected with a Niskin bottle at the CO Sites 2 and 4 as well as an area approximately 400 km west of the SSF (11°13.58’N, 139°15.18’E) (Table 1). POM in the seawater samples was subsampled on-board by a glass fiber filter (Advantec GC-5, the filter was heated at *c*. 450°C, 2h before using). The filters were preserved in a −80°C freezer until on-shore analyses. A filtrate at the reference site was used as a reference seawater for fluid chemistry.

#### Sediment

Sediment cores were obtained from inside (inside core) and at the edge (edge core) of a clam colony at CO Site 4 using a push corer (30 cm in length and 10 cm in diameter) during expedition YK13-08. Another sediment core was obtained as a reference (reference core) several kilometers away from the SSF (11°40.65’N, 142°59.26’E) (Table 1) during expedition YK14-13. Immediately after on-board recovery, the sediment cores were sliced horizontally at 3–5 cm intervals according to the sedimentary layers with different colors. Pore water was squeezed from about one-quarter of each sediment slice using a plastic syringe with a vice, following the procedures in ref. [26]. Another one-quarter of each sediment slice was provided for sulfide extraction on-board as described later. The remaining sediment was frozen at −80°C and taken back to an on-shore laboratory. An aliquot of the pore water was used for on-board analyses while the rest was filtered using a membrane filter (pore size = 0.45 µm) and stored in a refrigerator until further analyses. For methane analysis, 2 cm^3^ of sediments were subsampled with a sterilized cut syringe and were placed into 30 mL glass vials with 3 mL water containing 0.1 mL of saturated HgCl_2_ solution. Each vial was sealed with a Teflon-coated butyl-rubber septum with an aluminum seal and heated to 70°C for ~30 min. After cooling at room temperature, the methane concentration was determined on-board, as described below.

#### Chimney

Chimneys of the SSF have been classified into three types based on the mineralogy, the appearance (colors, spiky/tubular textures, and microbial mats), and the animal community inhabiting the surface [10]: (1) the Type I brucite-rich chimney has well developed microbial mats but a lack of densely-colonized animal, likely due to the relatively high flux of the highly alkaline and reduced fluid, (2) the Type II carbonate-rich chimney has obvious microbial mats and densely-colonized animals on it, the relatively lower fluid flux likely allows organisms to settle and flourish, and (3) the Type III brucite-poor chimney is dissolving and alternating after the fluid discharge has ceased.

In this study, eight brucite-carbonate chimneys were collected from the SSF CH Sites (Table 1). Two Type II chimneys were recovered from CH Site 1 (Chim 2) during expedition YK13-08 and CH Site 3 (Chim 4) during expedition YK14-13. Two Type I chimneys were also sampled from CH Site 4 (Chim 5 and 6) during expedition YK14-13. Each of these four chimneys was divided into four vertical sections: the root, lower, middle, and upper sections. Each section of the chimneys was further divided into inner and surfaces when possible. The middle part of the sample Chim 2 was lithified and sliced into round slices. These fragments of chimneys were preserved at −80°C for on-shore analyses. The pore waters of fragile parts of the chimneys (Chim 5 from CH Site 4 during expedition YK14-13, Chim 9 from CH Site 4 and Chim 10 from CH Site 3 during expedition YK15-11, and Chim 16 from CH Site 4 and Chim 18 from CH Site 3 during expedition KR16-14) were also collected by the same methods as for the sediment pore water. For methane analyses, ~5 cm^3^ of chimney fragments were subsampled from the inner part of the chimney structure with pore water and placed into 140 mL glass vials. When the chimney samples were massive and lithified, an agate mortar and pestle was used to crush the fragments into pebble-sized fragments (<5 mm in diameter) before placed into the vials. Each vial was sealed with a Teflon-coated butyl-rubber septum with an aluminum seal after adding 0.1 mL of saturated HgCl_2_ solution. The vials were kept at room temperature until on-shore analysis.

#### Seeping fluids

For on-site fluid sampling, we used a ‘D-WHATS’ sampler, a version of the WHATS-series gas tight fluid samplers [27, 28], and an all titanium fluid inlet with a temperature probe. D-WHATS is designed to resist pressure up to 100 MPa and has four 50 mL bottles made by high tensile titanium alloy. We attempted to collect discharging geofluids holding up the fluid inlet of the sampler just above the colonies (CO Sites 2 and 4) and the chimneys (CH Sites 1, 3, and 4) during the expeditions YK13-08, YK14-13, YK15-11, and KR16-14. Although no visual signature of fluid discharge was seen, a chemical signature suggesting the existence of seepage was detected (see *Results*). As a reference seawater sample, we used the filtrate after removal of the reference POM.

Since the fluid seepage was evidently very slow, we developed a seafloor-deployed fluid accumulator, named the proto-GATS (Geofluid Accumulating Trap System) marker (hereinafter, referred to as GATS marker), in order to accumulate the invisible but presumably discharging fluids on board in haste. The concept of the GATS marker is the displacement of pre-filled seawater by presumably buoyant (lower-density) deep-sourced seeping fluid. The GATS marker consists of a 40 cm-long transparent polycarbonate tube (generally used for push corer), a vinyl chloride plate for sealing one end of the tube fixed with stainless-steel screw nails and resin type adhesive, a buoyant marker attached to the outside of the plate for keeping the GATS marker vertical, and four stainless-steel chains at the open-ended side serving as ballasts. The expected sample volume of the GATS marker (~3,300 mL) is sufficient to fill the bottles (50 mL) and fluid path tubes of the D-WHATS (~100 mL). Two GATS markers (named GATS-blue and -red) were deployed on the base of Type II chimneys at CH Site 3 after the elongated chimneys were broken during the dive 6K#1433 in expedition YK15-11. During the dive KIV#709 in expedition KR16-14 (471 days after deployment), we identified whitish precipitations on the inner wall and edges of GATS-blue, whereas black precipitations were identified on the inner wall of GATS-red. For each GATS marker, approximately 1 L of the ~3 L fluid accumulated was carefully sampled by D-WHATS.

### Analytical methods

Analytical methods used in this study are listed in Table 2. Animal soft tissue samples for carbon and nitrogen isotope measurements were treated with 1 N HCl, followed by treatment with chloroform:methanol mixture (3:1 by volume) to remove carbonates [29] and lipids [30]. Then, they were lyophilized and pulverized. For sulfur isotope measurements, animal soft tissues were dialyzed repeatedly using 1M LiCl solution to remove excess seawater sulfates [31, 32]. All organic sulfur species in the dialyzed soft tissue were converted to sulfate using a Parr Bomb 1108 apparatus and then recovered as a BaSO_4_ precipitate in accordance with a previously reported methodology [31, 32].

**Table 2:** Analytical methods and estimated errors.

Analyte		Method	Accuracy	Ref.
**Fluid**	pH	pH meter	± 0.02	[33]
	Alkalinity	potentiometric titration	± 0.02 mM	[33]
	Si	colorimetric (molybdenum blue)	± 8%	[33]
	Na^+^, K^+^, Mg^2+^, Ca^2+^	ICP-OES	± 5%	
	NH_4_^+^	colorimetric (indophenol)	± 8%	[33]
	Cl^−^, Br^−^, SO_4_^2−^	ion chromatography	± 5%	
	NO_3_^−^	HPLC	± 5%	[34]
	H_2_S	precipitated as BaSO_4_ from HgS, gravimetric	± 5%	[35]
	CH_4_	GC-HID	± 10%	
**Sediment, Chimney, POM**	TOC, TN	EA (flush combusion method)	± 5%	
	AVS	precipptated as BaSO_4_ from CdS, gravimetric	± 5%	[36]
**Isotopic Ratio**	δ^13^C of CH_4_	CF-irMS	± 0.3%	[37]
	δ^13^C, δ^18^O of carbonate	irMS with dual inlet	± 0.06‰	
	δ^13^C, δ^15^N of sediment, chimney, POM, animal	CF-irMS	± 0.2, ± 0.3‰	
	All of δ^34^S	precipitate as BaSO_4_, CF-irMS	± 0.3‰	

Fluid pH, alkalinity, and concentrations of ammonium and silica of all fluid samples were measured on-board using pH electrode and colorimetric analyses as described by ref. [33]. Major cation (Na^+^, Mg^2+^, K^+^, and Ca^2+^) and anion (Cl^−^, Br^−^, and SO_4_^2−^) in the pore water and fluid samples were quantified with inductivity coupled plasma optical emission spectrometer (ICP-OES) and ion chromatography, respectively. The accuracies and precisions of ICP-OES and ion chromatography analyses were verified using IAPSO seawater. Nitrate concentrations were measured by HPLC [34] and calibrated using the CSK (The International Coordination Group for Cooperative Study of the Kuroshio and Adjacent Region) standard distributed by Wako Pure Chemical Industries, Ltd.

H_2_S concentrations of the fluid samples were quantified by the weight of HgS, which was precipitated by the addition of a HgCl_2_ solution. Analytical precisions of the H_2_S concentration were estimated to be within 5%. The precipitated HgS was converted to BaSO_4_ by adding inverse aqua regia (nitric acid:hydrochloric acid = 3:1, by volume), and subsequently bromine for dissolution and oxidation, following the method in ref. [35]. Sulfate ions dissolved in the fluids were also recovered as BaSO_4_ precipitates.

For total organic carbon (TOC) and total nitrogen (TN) measurements, frozen POM, sediment, and chimney samples were lyophilized. A 1 N HCl solution was added to remove carbonate before being dried *in vacuo* using NaOH pellets as a desiccant. To measure sulfide concentrations in sediment samples, a portion of the sediment was used for the extraction of acid volatile sulfide-sulfur (AVS) on-board, following the referenced procedures in ref. [36]. The AVS extracted as CdS precipitates was oxidized using a few drops of *c*. 30% hydrogen peroxide solution and converted into BaSO_4_ precipitate by the addition of a BaCl_2_ solution. The resulting BaSO_4_ precipitates and dried sediment fractions used for AVS extractions were precisely weighed, and sulfide-sulfur concentrations were calculated in mmol/kg dry sediment. The quantitative analytical error associated with the overall process of TOC, TN, and AVS determinations was less than 5%.

TOC and TN levels in the POM, sediment, and chimney samples and all carbon, nitrogen, and sulfur isotopic compositions of TOC, TN, the BaSO_4_ precipitates, glass fiber filters containing POM and animal samples were measured using EA/irMS. All the isotopic values are expressed using δ notation in permil deviation (‰) from international reference materials (VPDB: Vienna Pee Dee Belemnite for δ^13^C, atmospheric N_2_ for δ^15^N, and CDT: Cañon Diablo troilite for δ^34^S). Analytical errors associated with the overall process of these determinations were less than 5% for the quantitative analyses and less than ±0.2, ±0.3, and ±0.3‰ for δ^13^C, δ^15^N and δ^34^S isotopic compositions, respectively. Stable carbon and oxygen isotopic compositions of carbonate minerals of the chimneys were determined using an irMS with an automated carbonate reaction system. Analytical precision for carbon and oxygen isotope analyses was better than 0.06‰.

Methane concentrations of sediments and chimneys were determined using an in-house standard comprising 100 ppm CH_4_ over a He matrix (purity is 99.9999%: Iwatani Gasnetwork Corporation, Osaka, Japan). The measurement was performed on-board for sediment samples, and on-shore for chimney samples. The overall error rate for the analysis was expected to be within 10%. Carbon isotopic compositions were determined by continuous-flow isotope ratio mass spectrometry (CF-irMS) with an on-line gas preparation and introduction system connected with a mass spectrometer (for further details, see ref. [37]). The concentration was presented in mole abundance per cm cubic sediment/chimney volume (mol/cm^3^). As listed above, isotopic values are expressed using δ^13^C in permil deviation (VPDB‰). The analytical precision was estimated to be 0.3‰.

The TOC, TN, TOC/TN atomic ratio, and isotopic values of sediment, chimney, and animal samples were examined using mean comparison *t*-tests or one-way ANOVA in order to evaluate the statistical significance of the differences observed.

### Isotopic signatures of supposed basal food sources and evaluation of the proportion of each food source

Four basal food sources were supposed as the nutrient and energy sources of the animals in the SSF. Three of the four sources are chemosynthetic products expected from abundant sulfide and methane in environment, including the following types: 1. Thioautotrophic microbes using the Calvin-Benson-Bassham cycle as a carbon fixation pathway (hereinafter, referred to as thiotroph-CBB), 2. Thioautotrophic microbes using the reductive tricarboxylic acid cycle (thiotroph-rTCA), and 3. Methanotrophic microbes. Methanotrophs are generally regarded as heterotrophs [7], but in the present study we regard them as chemosynthetic microbes (unless otherwise noted). Fourth basal food source is photosynthetic products by marine phytoplankton near the sea surface.

The relative contributions of four basal food sources for the SSF animals can be evaluated by carbon and sulfur isotopes assuming isotope mass balance models [38] as followings:

$$\delta^{13}C_{x}=f_{tc}\delta^{13}C_{tc}+f_{tr}\delta^{13}C_{tr}+f_{mt}\delta^{13}C_{mt}+f_{pp}\delta^{13}C_{pp}$$ Eq. 1

$$\delta^{34}S_{x}=f_{tc}\delta^{34}S_{tc}+f_{tr}\delta^{34}S_{tr}+f_{mt}\delta^{34}S_{mt}+f_{pp}\delta^{34}S_{pp}$$ Eq. 2

$$f_{tc}+f_{tr}+f_{mt}+f_{pp}=1$$ Eq. 3

where, *f*, δ^13^C, and δ^34^S, are respectively the proportions and carbon and sulfur isotope ratios of each animal and sources represented by subscripts (x: animal examined; tc: thiotroph-CBB; tr: thiotroph-rTCA; mt: methanotroph; pp: photosynthetic production).

The δ^13^C value of the thiotroph-CBB which and -rTCA are known to be typically range between −40 to −30‰ [16, 39] and −15 to −10‰ [40, 41], respectively. The δ^13^C_tc_ and δ^13^C_tr_ values are assumed to be −35‰ and −12.5‰, respectively (Table 3). The δ^13^C_mt_ value generally reflects that of the methane assimilated [42]. The δ^13^C values of the methane at CO and CH Sites were determined by porewater analyses at −48‰ and −10‰ in CO and CH sites, respectively (see *Results*). The δ^13^C_pp_ values has been reported in a range from *c*. −25 to *c*. −20‰ [38, 43−45]. The δ^13^C value of seawater POM at the reference site was in fact −24.6‰ (see *Results*). The δ^34^S value s of thiotrophs (both δ^34^S_tc_ and δ^34^S_tr_) are assumed to be identical to that of hydrogen sulfide utilized because of the known little isotope effect during assimilation [19]. The δ^34^S values of methanotrophs and photosynthetic products (δ^34^S_mt_ and δ^34^S_pp_) typically range from *c*. +15 to *c*. +21‰ (c.f. [17]) as well as seawater sulfate (+21‰ [46]). The sulfur isotope mass balance (Eq. 2) can be approximated as a following:

**Table 3:** The isotopic compositions of endmembers for estimation of the proportion of each basal food source.

Basal source	δ^13^C (‰)	reported value (‰)	δ^34^S (‰)	reported value (‰)	δ^15^N (‰)	reported value (‰)	Ref.
Thiotroph-CBB (CO Site)	−35	−40 to −30	−10	that of H_2_S	n.d.	<0 to +5	[16, 17, 19, 39], This study
Thiotroph-CBB (CH Site)	−35	*ditto*	+24	*ditto*	n.d.	*ditto*	*ditto*
Thiotroph-rTCA (CO Site)	−12.5	−15 to −10	−10	*ditto*	n.d.	*ditto*	[17, 40, 41], This study
Thiotroph-rTCA (CH Site)	−12.5	*ditto*	+24	*ditto*	n.d.	*ditto*	*ditto*
Methanotroph (CO Site)	−48	that of CH_4_	+20	+16 to +21	n.d.	*ditto*	[17, 42], This study
Methanotroph (CH Site)	−15	*ditto*	+20	*ditto*	n.d.	*ditto*	*ditto*
Photosynthetic POM	−24.6	−25 to −20	+20	*ditto*	+7.5	+3 to +10	[38, 43–48], This study

n.d.; not determined.

$$\delta^{34}S_{x}=\left(f_{tc}+f_{tr}\right)\delta^{34}S_{sulfide}+\left(f_{mt}+f_{pp}\right)\delta^{34}S_{sulfate}$$ Eq. 4

The δ^34^S_sulfide_ at CO and CH sites were respectively assumed to be −10‰ (identical with AVS in shallow sediment) and +24‰ (identical with H_2_S in fluid) (see *Results*). The δ^34^S_sulfate_ was assumed to be +20‰ [46]. Because there are four variables in these three equations (Eqs. 1, 3, and 4), the proportions (*f*) cannot be constrained in general. In the case of animals which have δ^34^S values identical to δ^34^S_sulfide_ or δ^34^S_sulfate_, the sulfur sources can be constrained to be sulfide (*f*_tc_ + *f*_tr_ = 1) or sulfate (*f*_mt_ + *f*_pp_ = 1), respectively, from Eqs. 3 and 4.

The δ^15^N value of the photosynthetic product has been reported in a range from *c*. +3 to *c*. +10‰ [47, 48], and the seawater POM at the reference site had δ^15^N = +7.5‰ (see *Results*). On the other hand, the δ^15^N values of chemoautotrophs and methanotrophs, probably controlled by δ^15^N values of their nitrogen sources and isotope effects on assimilation, are still poorly constrained [49, 50]. Nonetheless, δ^15^N values of organic matter including animal tissues are not higher than +7.5‰, photosynthetic product value, indicating significant contribution of chemosynthetically-produced nitrogen nutrient as basal food source because δ^15^N value increases along with increasing trophic level.

## Results

### Fluids and pore waters

The chemical compositions of fluid samples collected just above the seafloor at the CO and CH Sites by the D-WHATS sampler were almost identical to those of the reference seawater sample (Table 4). On the other hand, the two fluid samples collected from the GATS markers (GATS-red and -blue fluid) showed distinct fluid chemistry from the ambient seawater. The GATS-blue fluid exhibited a significantly high pH (9.89), and low sulfate (7.6 mM) and Mg^2+^ (7.4 mM) concentrations. The sulfate and Mg^2+^ depletions were also significant in the GATS-red fluid but the magnitude of depletion was smaller. In addition, the pH value of the GATS-red fluid sample was lower than that of the reference seawater.

**Table 4:** Chemical and isotopic compositions of water samples analyzed.

**Sample name**	SUP#_Dive#-ID#	Site	Sample information	pH	alk.	Si	Na^+^	K^+^	Mg^2+^	Ca^2+^	NH_4_^+^	Cl^−^	Br^−^	NO_3_^−^	SO_4_^2−^	δ^34^S (SO_4_)	H_2_S	δ^34^S (H_2_S)	CH_4_	δ^13^C (CH_4_)
					(meq/L)	(μM)	(mM)	(mM)	(mM)	(mM)	(μM)	(mM)	(mM)	(μM)	(mM)	(‰)	(mM)	(‰)	(μmol/g)	(‰)
**Fluids**																				
	12541–12001_6K1365-DW2	CO Site 2	Above inside of colony	7.7	2.4	134.6	473	10.28	51.9	10.50	<0.1	547	0.86	n.a.	28.4	n.a.	n.d.	n.a.	n.a.	n.a.
	2541–14001_6K1403-DW	CO Site 2	Above inside of colony	7.8	2.4	136.9	482	10.27	56.4	9.93	<0.1	558	0.90	n.a.	28.8	n.a.	n.d.	n.a.	n.a.	n.a.
	12541–12001_6K1362-DW4	CO Site 4	Above inside of colony	7.8	2.2	130.5	454	9.89	50.3	10.02	<0.1	532	0.85	n.a.	27.7	n.a.	n.d.	n.a.	n.a.	n.a.
	12541–12001_6K1362-DW2	CO Site 4	Above inside of colony	7.8	2.3	131.4	440	9.52	49.3	9.74	<0.1	518	0.84	n.a.	27.4	n.a.	n.d.	n.a.	n.a.	n.a.
	12541–12001_6K1365-DW4	CH Site 1	Near a chimney root	7.8	2.5	127.3	474	10.20	52.1	10.54	0.39	538	0.84	n.a.	28.2	n.a.	n.d.	n.a.	n.a.	n.a.
	12541–16002_KIV708-DW2	CH Site 3	Near bottom of chimney	7.83	n.a.	140.3	473	10.10	52.9	10.32	<0.1	518	n.a.	30.5	27.8	n.a.	n.d.	n.a.	n.a.	n.a.
	12541–15002_6K1433-DW2	CH Site 3	On edge of broken chimney	7.87	3.0	140.4	455	10.19	53.1	10.39	n.a.	486	0.85	31.7	25.3	n.a.	n.d.	n.a.	n.a.	n.a.
	12541–15002_6K1433-DW4	CH Site 4	On edge of broken chimney	7.80	2.7	139.8	469	10.30	53.6	10.44	n.a.	505	0.86	29.2	26.1	n.a.	n.d.	n.a.	n.a.	n.a.
	12541–14001_6K1397-N	*c*. 400 km away from SSF	For reference	7.80	2.1	132.2	482	10.16	58.4	10.43	<0.1	558	0.86	n.a.	28.6	n.a.	n.d.	n.a.	n.a.	n.a.
**GATS fluids**																				
GATS-red fluid	12541–16002_KIV709-DW2	CH Site 3	In GATS maker (red)	7.31	2.2	99.2	466	10.08	42.3	10.37	11.7	511	n.a.	30.7	24.2	+20.3	n.d.	n.a.	n.a.	n.a.
GATS-bleu fluid	12541–16002_KIV709-DW4	CH Site 3	In GATS maker (bleu)	9.89	0.4	21.0	495	10.41	7.4	10.92	53.2	577	n.a.	10.1	7.6	+20.4	1.33	+24.2	n.a.	n.a.
**Pore waters**																				
Inside core	12541–12001_6K1362-H2	CO Site 4	Inside of a clam colony																	
			Above seawater	7.8	n.a.	n.a.	473	10.55	52.8	10.57	n.a.	595	0.84	n.a.	30.3	n.a.	n.a.	n.a.	n.a.	n.a.
			At 2.5 cm sediment depth	7.7	n.a.	237.3	474	12.98	51.4	10.34	23.56	544	0.85	6.4	28.0	n.a.	n.a.	n.a.	n.a.	n.a.
			At 7.5 cm sediment depth	8.0	n.a.	n.a.	396	13.62	40.4	8.07	<0.1	541	0.89	n.a.	27.0	n.a.	n.a.	n.a.	5.9	−48.0
			At 12.5 cm sediment depth	8.2	n.a.	210.0	502	13.46	49.1	9.78	9.38	573	0.86	n.a.	26.8	n.a.	n.a.	n.a.	19.7	−47.4
			At 16 cm sediment depth	8.2	n.a.	204.1	508	12.99	51.5	10.04	8.47	571	0.86	n.a.	26.7	n.a.	n.a.	n.a.	19.7	−48.3
Edge core	12541–12001_6K1362-H5	CO Site 4	Edge of a clam colony																	
			At 2.5 cm sediment depth	7.5	n.a.	225.7	492	12.30	51.6	10.55	15.79	547	0.88	n.a.	27.7	n.a.	n.a.	n.a.	1.2	<d.l.
			At 7.5 cm sediment depth	7.5	n.a.	218.5	489	12.19	50.8	10.35	10.52	567	0.85	n.a.	28.6	n.a.	n.a.	n.a.	1.0	<d.l.
			At 11 cm sediment depth	8.0	n.a.	231.2	500	13.35	50.3	10.39	<0.1	580	0.89	n.a.	28.8	n.a.	n.a.	n.a.	2.0	<d.l.
Reference core	12541–15002_6K1432-H3	*c*. 5 km away from SSF	For reference																	
			At 1.5 cm sediment depth	7.9	n.a.	n.a.	491	11.60	51.9	10.55	n.a.	577	0.87	n.a.	29.2	n.a.	n.a.	n.a.	n.a.	n.a.
			At 4.5 cm sediment depth	7.9	n.a.	n.a.	469	11.80	50.4	10.42	n.a.	562	0.87	n.a.	28.2	n.a.	n.a.	n.a.	n.a.	n.a.
			At 8 cm sediment depth	7.8	n.a.	n.a.	484	11.79	51.4	10.66	n.a.	534	0.87	n.a.	27.0	n.a.	n.a.	n.a.	n.a.	n.a.
Chim 10*	12541–15002_6K1433-R05	CH Site 3	Squeezed and extracted from Type II chimney	n.a.	n.a.	2.3 ~ 8.6	419 ~ 449	10.14 ~ 10.40	2.8 ~ 3.1	7.47 ~ 9.07	n.a.	517 ~ 544	1.04 ~ 1.07	20.4 ~ 38.8	1.5 ~ 2.7	n.a.	n.a.	n.a.	0.1 ~ 0.4	−10.3 ~ −8.6
Chim 16	12541–16002_KIV708-R06	CH Site 3	Squeezed and extracted from Type II chimney	10.00	3.3	0.6	511	10.99	0.3	9.46	44.2	546	n.a.	13.6	5.6	n.a.	n.a.	n.a.	0.01	−18.1
Chim 5*	12541–14001_6K1404-R05	CH Site 4	Squeezed and extracted from Type I chimney	8.6 ~ 9.3	1.4 ~ 1.8	2.1 ~ 9.2	481 ~ 498	10.12 ~ 14.63	54.2, 55.2	10.84 ~ 12.56	4.7 ~ 11.7	555 ~ 569	0.83 ~ 0.85	n.a.	27.2 ~ 28.7	n.a.	n.a.	n.a.	0.04	−21.5
Chim 9*	12541–15002_6K1433-R04	CH Site 4	Squeezed and extracted from Type I chimney	n.a.	n.a.	1.7 ~ 10.0	366 ~ 432	9.68 ~ 11.60	2.0 ~ 8.6	6.98 ~ 17.10	n.a.	500 ~ 549	1.49 ~ 1.52	38.4 ~ 48.0	6.2 ~ 9.8	n.a.	n.a.	n.a.	0.01 ~ 0.1	−16.4 ~ −15.2
Chim 18	12541–16002_KIV708-C01	CH Site 4	Squeezed and extracted from Type I chimney	9.18	n.a.	4.9	468	10.09	36.0	20.23	2.8	630	n.a.	27.6	20.0	n.a.	n.a.	n.a.	n.a.	n.a.

n.a.; not analyzed

n.d.; not detected

d.l.; detection limit

*; Chimney ID listed in Okumura et al., 2016a [10]

δ^34^S values of sulfate (+20.3 and +20.4‰) in the GATS markers were almost identical to those of the ambient seawater (*c*. +21‰ [46]). The alkaline GATS-blue fluid contained a significant amount of H_2_S (1.3 mM) with higher δ^34^S value (+24.2‰) than the seawater sulfate δ^34^S value.

**Fig 2:**
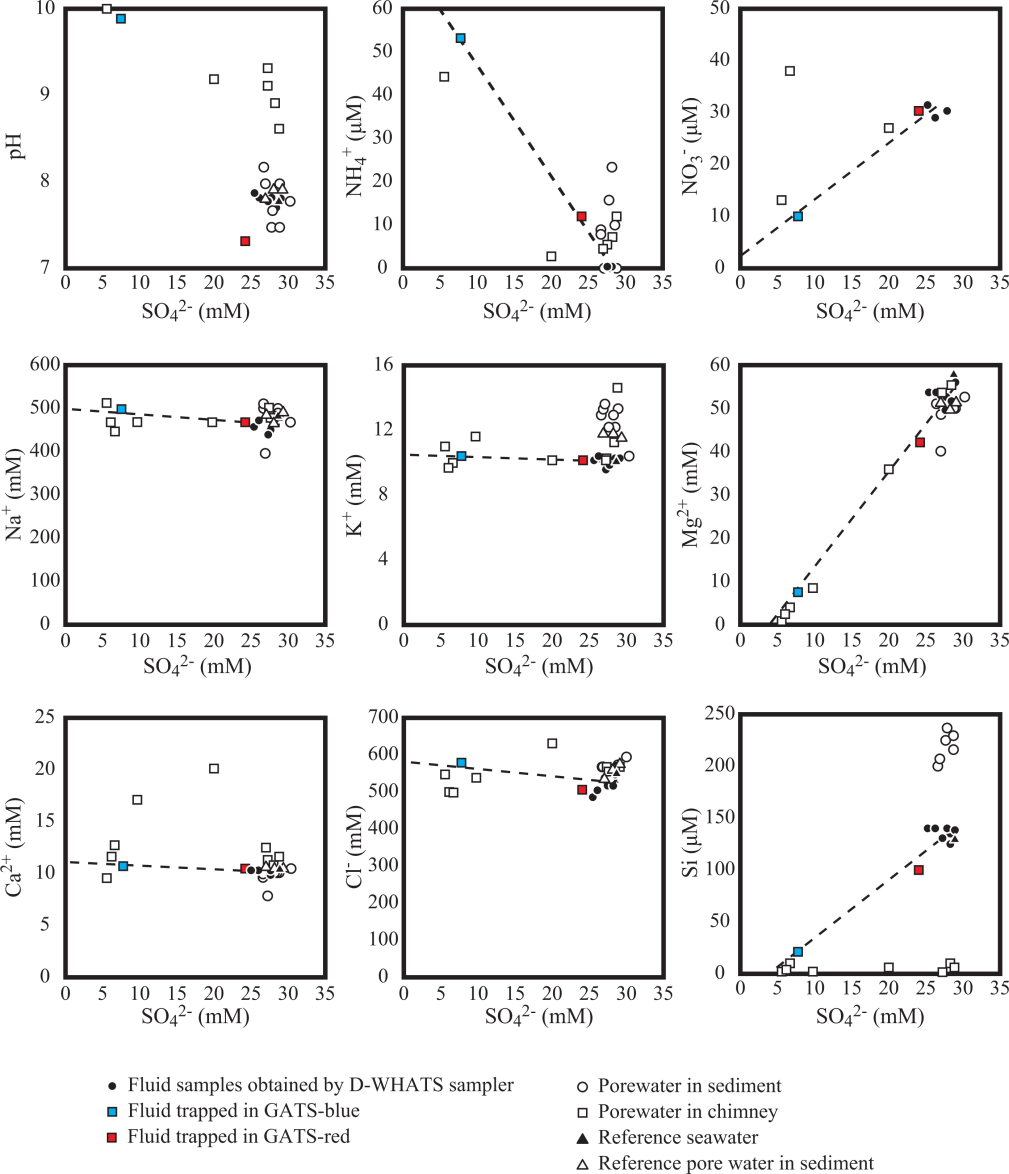
Plots of sulfate concentrations versus each component of the studied water samples. Filled circles represent samples collected from around the clam colonies and near the chimneys in the SSF. Filled black squares represent the results of the fluid samples, excluding the GATS fluids. Filled red and blue squares represent the results of GATS-red and -blue fluids, respectively. Open circles and squares represent the pore water samples squeezed from sediments beneath the clam colony and the brucite-carbonate chimneys, respectively. Dashed lines represent a plausible mixing line between fluid samples containing GATS fluids and the ambient seawater.

Fig 2 shows plots of major ions versus sulfate concentrations in all the fluid and pore water samples. Concentrations of some major ions, such as Na^+^, K^+^, Ca^2+^, and Cl^−^, were nearly constant against the changes in sulfate concentrations. On the other hand, Mg^2+^ and Si concentrations in some fluid samples were significantly lower than those in the reference seawater.

Chemical compositions of pore water obtained from the inside and the edge core in CO Site 4 were almost identical to the reference core with exceptions of sulfate and methane (Table 4). Sulfate concentrations in the pore water acquired from the deep layer (>10 cm below sea floor (cmbsf)) of the inside core were slightly lower (26.7–26.8 mM) than those from the edge core (28.6–28.8 mM) and also the reference core (27.7–28.8 mM). Methane concentrations of the clam colony sediment were approximately ten times higher inside the colony (5.9–19.7 μmol/cm^3^ sediment) than on the edge region (1.0–2.0 μmol/cm^3^ sediment; Table 4). Methane-enriched deeper sediment layers of the inside core (7.5, 12.5 and 16 cmbsf) showed δ^13^C values between −48.3 to −47.4‰. On the other hand, methane-depleted chimney pore waters (<0.5 μmol/cm^3^ chimney fragment) showed higher δ^13^C values ranging from −21.5 to −8.6‰ (Table 4).

Although chemical compositions of pore waters retrieved from porous chimneys were variable, they were generally characterized by high pH and low concentrations of silica and sulfate when compared to the reference seawater level. Mg^2+^ and Ca^2+^ concentrations in the chimney pore waters exhibited a wide variation (0.3–55.2 mM and 6.98–20.23 mM, respectively).

### POM, sediments, and chimneys

The TOC (50.071–0.404 mg-C/L) and TN (0.007–0.069 mg-N/L) levels of POM in seawater just above the clam colonies were higher than those of the reference site (0.031 mg-C/L and 0.002 mg-N/L). δ^13^C (−29.4 to −27.3‰) and δ^15^N values (+5.2 to +5.7‰) of the colony-water POM were both lower than those of the reference POM (δ^13^C = −24.6‰ and δ^15^N = +7.5‰). The δ^13^C and δ^15^N value of the reference POM fell into the range of typical oceanic phytoplankton (δ^13^C = *c*. −25 to *c*. −20‰ and δ^15^N = *c*. +3 to *c*. +10‰ [38, 43−45, 47, 48]) (Table 5). Sulfur isotope ratio of the POM samples cannot be determined due to low sulfur content.

**Table 5:** Chemical and isotopic compositions of POM samples.

Sample name	SUP#_Dive#-ID#	Site	Sample information	Water Depth	TOC	δ^13^C	TN	δ^15^N	TOC/TN
				(m)	(mg-C/L)	(‰)	(mg-N/L)	(‰)	(atomic ratio)
colony-water POM	12541–14001_6K1403-N	CO Site 2	Above a colony	5618	0.325	−29.4	0.034	+5.7	11.2
	12541–12001_6K1365-NG	CO Site 2	On edge of a colony	5623	0.404	−28.7	0.069	+5.2	6.8
	12541–12001_6K1362-NG	CO Site 4	Above a colony	5633	0.071	−28.0	0.007	+5.5	11.8
	12541–12001_6K1362-NR	CO Site 4	obtained 30 m away from a colony	5825	0.021	−27.3	0.001	n.a.	24.5
reference POM	12541–14001_6K1397-N	*c*. 400 km away from the SSF	For reference	4640	0.031	−24.6	0.002	+7.5	23.2

n.a.; not analyzed

**Fig 3:**
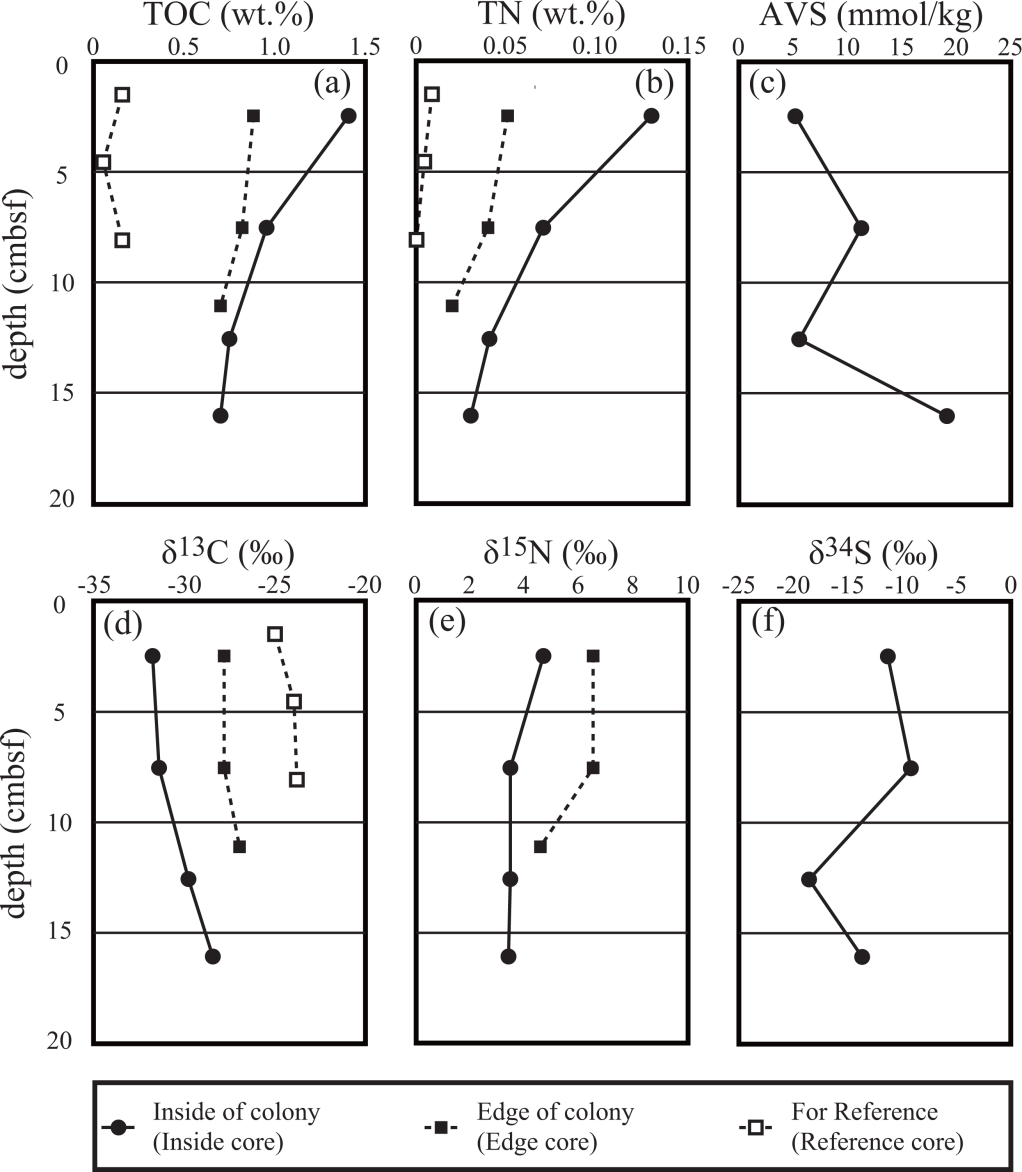
Vertical profiles of the chemical and isotopic compositions obtained from sediment samples taken from inside and the edge of a clam colony in CO Site 4. Upper plots show levels of TOC (a), TN (b), and AVS (c) in the sediments. The lower plots (d, e, and f) show each isotopic composition corresponding to the upper plots. Solid circles and squares represent samples taken from the inside and edge of the clam colony, respectively. Open squares represent the reference core sample.

The TOC and TN levels of the inside and edge cores at CO Site 4 were higher at shallower part in each core and obviously higher than those of the reference core through the depth (t-test, *p* < 0.05 in TOC and TN of both cores and reference core). Notably, the TOC level in the shallow layer (<5 cmbsf) of the inside core was greater than 1 wt%. δ^13^C values of TOC in both cores were lowest in the surface TOC-enriched layer (−31.8‰) and increased with depth to −27.0‰. In turn, δ^13^C values of TOC in the reference core were −25.0 to −23.8‰ and identical to that of the reference seawater POM (Table 6 and Fig 3). AVS levels were 5.1−19.1 mM-S/kg in the inside core while not detectable (<0.3 mM-S/kg) in the other cores. δ^34^S values of AVS of the inside core ranged from −18.6 to −9.2‰. As benthic biological community likely relies on nutrients in the sediment surface (<10 cmbsf) rather than the deep subseafloor (>10 cmbsf), δ^34^S_sulfate_ in Eq. 2 for CO Site is assumed to be −10‰, an averaged δ^34^S value at the sediment surface (0–10 cmbsf).

**Table 6:** Chemical and isotopic compositions of sediment samples obtained from inside and the edge of the clam colony (CO Site 4) and the reference site.

**Sample name**	SUP#_Dive#-ID#	Site	Sample information	TOC	δ^13^C	TN	δ^15^N	TOC/TN	AVS	δ^34^S
				(wt.%)	(‰)	(wt.%)	(‰)	(atomic ratio)	(mM-S/kg dry sed.)	(‰)
Inside core	12541–12001_6K1362-H2	CO Site 4	Inside of a clam colony							
			At 2.5 cm sediment depth	1.41	−31.8	0.13	+4.7	12.7	5.12	−11.3
			At 7.5 cm sediment depth	0.96	−31.4	0.07	+3.5	16.0	10.99	−9.2
			At 12.5 cm sediment depth	0.75	−29.8	0.04	+3.5	21.9	5.60	−18.6
			At 16 cm sediment depth	0.70	−28.5	0.03	+3.4	27.2	19.11	−13.6
Edge core	12541–12001_6K1362-H5	CO Site 4	At 2.5 cm sediment depth	0.89	−27.8	0.05	+6.5	20.8	n.d.	n.a.
			At 7.5 cm sediment depth	0.82	−27.8	0.04	+6.5	23.9	n.d.	n.a.
			At 11 cm sediment depth	0.70	−27.0	0.02	+4.6	40.8	n.d.	n.a.
Reference core	12541–15002_6K1432-H3	*c*. 5 km away from the SSF	for reference							
			At 1.5 cm sediment depth	0.16	−25.0	0.009	n.a.	20.7	n.d.	n.a.
			At 4.5 cm sediment depth	0.05	−23.9	0.004	n.a.	14.6	n.d.	n.a.
			At 8 cm sediment depth	0.16	−23.8	0.001	n.a.	186.7	n.d.	n.a.

n.a.; not analyzed

n.d.; not detected

The TOC and TN levels of a Type I chimney sample from CH Site 4 (Chim 6) were higher at the chimney surface than the inner part (t-test, *p* < 0.05 for TOC and TN in surface and inner) (Table 7). δ^13^C values of the inner part TOC showed a narrow range from −27.3 to −26.0‰, whereas those of the surfaces TOC varied widely from −30.3 to −22.8‰. δ^15^N values of TN in the inner part (−1.6 to +11.4‰) were similar to or higher than those of the surface (−3.6 to −1.4‰) (t-test, *p* < 0.01). Chimney surface of another Type I chimney sample from CH Site 4 (Chim 5) had similar TOC, TN, δ^13^C, and δ^15^N values of the surface part of the sample Chim 6 (Table 7).

**Table 7:** Analytical results of chemical and isotopic compositions of brucite-carbonate chimneys in the SSF.

**Sample name**	SUP#_dive#-ID#	Type*	Site	Part	Carbonate*		Organic matter				
					δ^13^C	δ^18^O	TOC	δ^13^C	TN	δ^15^N	TOC/TN
					(‰)	(‰)	(wt.%)	(‰)	(wt.%)	(‰)	(atomic ratio)
Chim 6*	12541–14001_6K1404-R05	I	CH Site 4	Inner of the root section	n.a.	n.a.	0.012	−26.0	0.001	+6.3	23.8
				Inner of the lower section	+23.6	+2.8	0.019	−26.3	0.001	+7.5	22.2
				Inner of the lower section			0.026	−27.3	0.002	+11.4	15.2
				Inner of the lower section			0.011	−27.2	0.001	+5.5	13.8
				Inner of the middle section	+13.9	+3.1	0.016	−26.3	0.001	+3.9	28.5
				Inner of the upper section	+12.7	+3.4	0.014	−26.4	0.001	−1.6	15.0
				Surface of the root section	n.a.	n.a.	0.055	−23.5	0.007	−3.6	9.5
				Surface of the lower section	+23.6	+2.8	0.084	−29.7	0.012	−1.4	7.9
				Surface of the middle section	+13.9	+3.1	0.047	−30.3	0.007	−2.2	7.5
				Surface of the upper section	+12.7	+3.1	0.046	−25.4	0.006	−2.3	8.8
Chim 5*	12541–14001_6K1404-R04	I	CH Site 4	Surface of the root section	−1.5	+3.1	0.072	−22.8	0.012	−2.8	7.0
				Surface of the middle section	−8.8	+3.4	0.144	−29.3	0.028	−2.3	6.0
				Surface of the upper section	−0.3	+5.2	0.026	−28.0	0.006	−3.7	5.2
Chim 4*	12541–14001_6K1402-R01	II (porous)	CH Site 3	Inner of the root section	+0.5	+3.7	0.026	−13.5	0.003	−4.7	10.1
				Inner of the root section	n.a.	n.a.	0.019	−19.0	0.002	−2.5	11.1
				Inner of the root section	n.a.	n.a.	0.255	−31.5	0.053	−2.3	5.6
				Surface of the chimney with tube worm	−0.3	+3.7	0.156	−16.0	0.026	−2.7	7.0
				Surface of the chimney with decomposed shell of tube worms	+0.1	+3.7	0.170	−14.2	0.022	−3.9	9.0
				Surface of the root section	n.a.	n.a.	0.471	−29.5	0.100	−2.0	5.5
Chim 2*	12541–12001_6K1365-R01	II (lithified)	CH Site 1	Inner (center of the chimney) of the middle section	+22.3	+4.0	0.043	−31.5	0.003	+0.4	16.7
				Inner (1 cm from the center) of the middle section	+19.7	+4.0	0.032	−32.3	0.002	−0.4	18.7
				Inner (2 cm from the center) of the middle section	+13.6	+3.8	0.033	−35.9	0.002	0.0	19.3
				Inner (3 cm from the center) of the middle section	+22.4	+3.6	0.024	−33.8	0.002	+3.0	14.0
				Inner (4 cm from the center) of the middle section	+19.1	+3.4	0.016	−32.3	0.001	+1.3	18.7
				Surface (5 cm from the center) of the middle section	+11.3	+3.2	0.053	−20.2	0.007	+8.0	8.8
				Surface (6 cm from the center) of the middle section	+2.9	+5.3	0.089	−18.8	n.a.	+3.5	n.a.

n.a.; not analyzed

*; Chimney ID listed in Okumura et al., 2016a [10]

Each of TOC, TN, δ^13^C and δ^15^N values of the porous Type II chimney sample from CH Site 3 (Chim 4) was comparable between surface and inner parts (Table 7). TOC and TN levels of the Type II chimney (both inner and surface) were 0.019−0.471 wt% and 0.002−0.100 wt%, respectively, and were higher (up to ten times) than Type I chimneys (Table 7). δ^13^C values of the chimney varied widely from −31.5 to −13.5‰, whereas δ^15^N values showed a narrow range from −3.6 to −1.4‰. A lithified Type II chimney sample from CH Site 1 (Chim 2) had lower TOC and TN levels and lower δ^13^C and δ^15^N values at the inner parts were lower than the surfaces.

Carbon and oxygen isotopic compositions of carbonate minerals were obtained from the four chimney samples (Chim 2, 4, 5, and 6) (Table 7). δ^18^O values (+3.5 ± 1.2‰) were uniform among all chimney carbonates, while δ^13^C values were highly varied from −8.8 to +23.6‰.

### Animals in CO Sites

Stable isotopic compositions of gill tissues of *C*. *mariana* from the CO Sites 2, 4 and 7 were not significantly different (one-way ANOVA, *p* > 0.05 for δ^13^C, δ^15^N and δ^34^S values). The isotope ratios obtained in this study (δ^13^C = −35.0 ± 1.1‰, δ^15^N = +3.3 ± 1.0‰, and δ^34^S = −10.7 ± 3.0‰; mean ± SD; n = 9; Table 8) were almost identical to the values from the CO Sites 1 and 2 reported previously (δ^13^C = −34.6 ± 0.5‰, δ^15^N = +4.2 ± 1.4‰; n = 3, and δ^34^S values were not reported [9]). Among tissues of the clams, the δ^15^N values of gill tissues were *c*. 3‰ lower those of the other tissues (Table 8). This difference of δ^15^N values between the gill and the other tissues of the clam has been observed also in other chemosynthetic communities (e.g. [51–54]), and interpreted as TN of gill tissue is occupied by symbiotic microbial cells and not representative to the host clams body. The clams and frenulate tubeworms had δ^34^S values of −10.7 ± 3.0‰ and −10.5‰, respectively, that were similar to those of AVS values (−10‰) in the associated shallow sediments. δ^13^C values of the frenulate tubeworms (−22.2 and −20.6‰) were much higher than those of the clams (t-test, *p* < 0.01).

**Table 8:** Stable carbon, nitrogen, and sulfur isotopic compositions of animal tissues of organisms collected from CO and CH Sites in the SSF.

**Sampling Site**	Fauna	Organ	δ^13^C	n	δ^15^N	n	δ^34^S	n
			(‰)		(‰)		(‰)	
CO Site 2	*Calyptogena mariana*	Gill	−35.3 ± 0.7	3	+2.6 ± 1.4	3	−8.8 ± 0.8	3
		Foot	−32.3 ± 0.4	3	+5.0 ± 0.6	3	−3.6, −8.8	2
		Mantle	−32.6 ± 0.5	3	+3.4 ± 3.1	3	+0.6 ± 3.2	3
		Adductor	−32.8 ± 1.2	3	+5.5 ± 1.3	3	−10.1, -7.9	2
	frenulate tubeworm	Bulk	−22.2, −20.6	2	+4.4, +2.1	3	−10.5, −10.5	2
		Tube	−21.6	1	+1.5	1	−9.0	1
	lumbrinerid polychaete	Bulk	−26.1	1	+4.9	1	+19.8	1
	amphipod	Bulk	−24.6 ± 1.0	3	+7.7 ± 3.0	3	+17.6, +15.5	2
	nematode	Bulk	−25.3	1	+10.4	1	n.a.	
CO Site 4	*Calyptogena mariana*	Gill	−35.1 ± 1.9	3	+3.4 ± 1.0	3	−9.2 ± 1.7	3
		Foot	−33.2 ± 0.6	3	+6.0 ± 2.0	3	−7.1, −7.0	2
		Mantle	−33.1 ± 0.5	3	+6.2 ± 0.9	3	−5.3, −2.0	2
		Adductor	−32.5 ± 0.6	3	+7.4 ± 0.9	3	−8.8, −9.0	2
	nautiliniellid polychaete	Bulk	−33.4 ± 0.8	9	+11.7 ± 0.7	9	−9.0 ± 1.1	6
	maldanid polychaete	Bulk	−31.0 ± 0.2	3	+10.8 ± 0.6	3	+2.2 ± 1.0	3
	eunicid polychaete	Bulk	−31.7	1	+12.4	1	+17.2	1
	*Bayerius* cf. *arnoldi*	Bulk	−32.3 ± 0.9	3	+8.8 ± 0.8	3	+0.5, +0.2	2
	amphipod	Bulk	−31.6 ± 1.5	3	+10.7 ± 1.6	3	+2.8	1
	isopod	Bulk	−33.3 ± 0.9	3	+7.1 ± 0.1	3	+1.9	1
	anthozoan	Bulk	−29.2 ± 0.9	4	+9.2 ± 0.5	4	−3.9 ± 1.2	4
CO Site 7	*Calyptogena mariana*	Gill	−34.5 ± 0.4	3	+3.9 ± 0.1	3	−13.9 ± 3.0	3
		Foot	−33.3 ± 0.6	2	+6.9 ± 0.4	3	−9.9	1
		Mantle	−33.5 ± 0.4	3	+6.0 ± 1.5	3	−7.1 ± 7.6	3
		Adductor	−33.4 ± 0.5	3	+7.5 ± 0.5	3	−14.6 ± 0.7	3
CH Site 1	*Phyllochaetepterus* polychaete	Bulk	−25.3	1	−1.3	1	+21.6	1
	polynoid polychaete	Bulk	−25.7, −25.4	2	+6.8, +5.9	2	+17.7, +10.8	2
	lumbrinerid polychaete	Bulk	−28.7, −28.0	2	+5.4, +1.9	2	+17.3, +18.3	2
	eunicid polychaete	Bulk	−24.6	1	+5.6	1	n.a.	
	*Bayerius* cf. *arnoldi*	Bulk	−25.0	1	+6.3	1	n.a.	
	amphipod	Bulk	−26.1 ± 2.8	3	+5.1 ± 1.8	3	+14.7 ± 2.3	3
	leptostracan	Bulk	−24.5	1	+7.2	1	n.a.	
	anthozoan	Bulk	−22.3	1	+0.3	1	+21.2	1
	nematode	Bulk	−23.5	1	+8.0	1	n.a.	
CH Site 3	*Phyllochaetepterus* polychaete	Bulk	−25.6	1	+1.7	1	+20.5	1

n.a.; not analyzed

**Fig 4:**
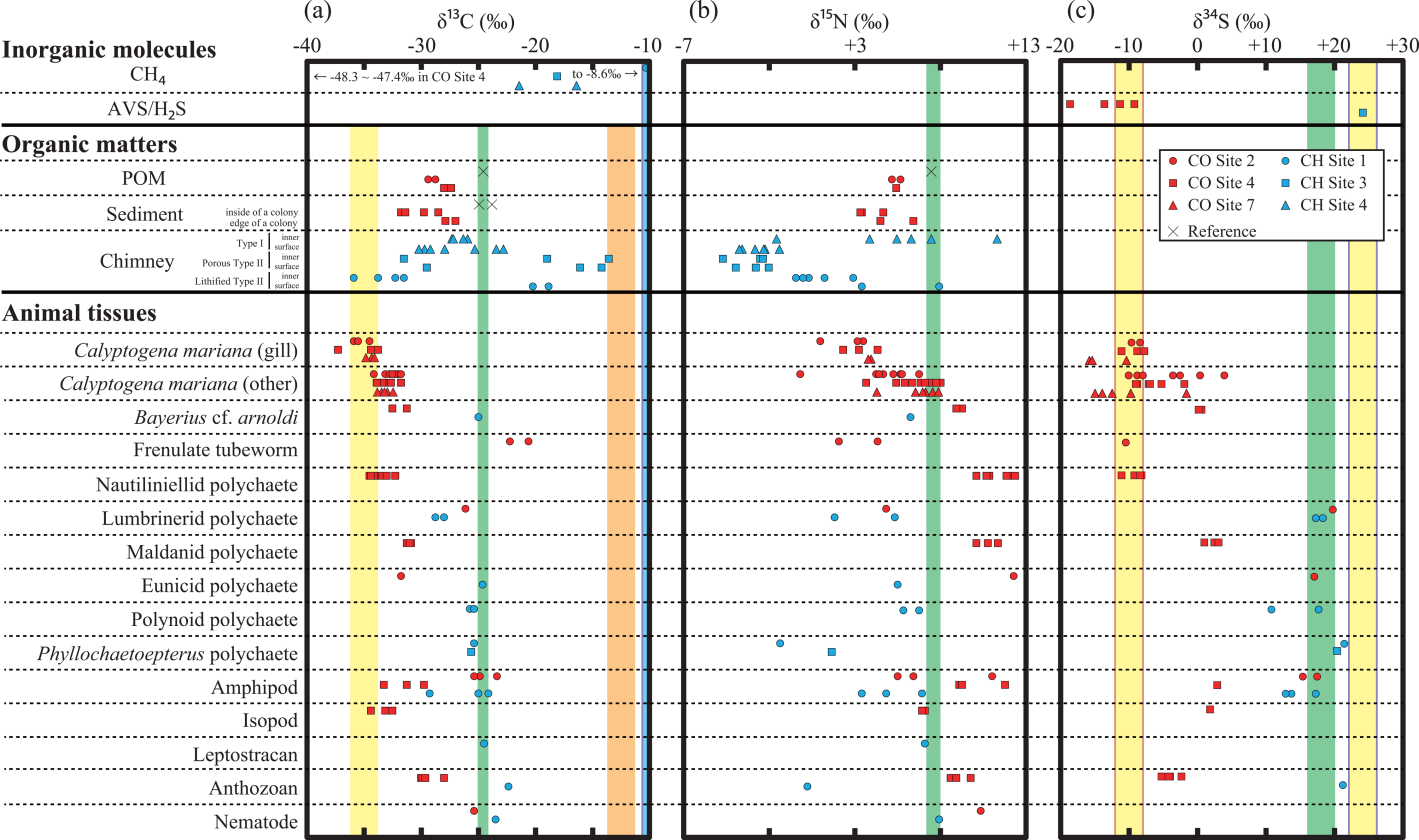
Compilation of the carbon (a), nitrogen (b), and sulfur (c) isotopic compositions analyzed in this study. In (a) and (b), yellow, orange, blue, and green shadows represent the expected ranges of the isotopic compositions of thiotroph-CBB, thiotroph-rTCA, methanotroph in CH Sites, photosynthetic organic matter (Table 3). The ranges of methanotroph in CO Sites are not shown. In (c), green shadow represents the expected ranges of the δ^34^S values of photosynthetic organic matter and methanotroph (δ^34^S_sulfate_) and yellow shadows with red and blue lines, respectively, represent the δ^34^S values of thiotrophs (δ^34^S_sulfide_) in CO and CH Sites.

**Fig 5:**
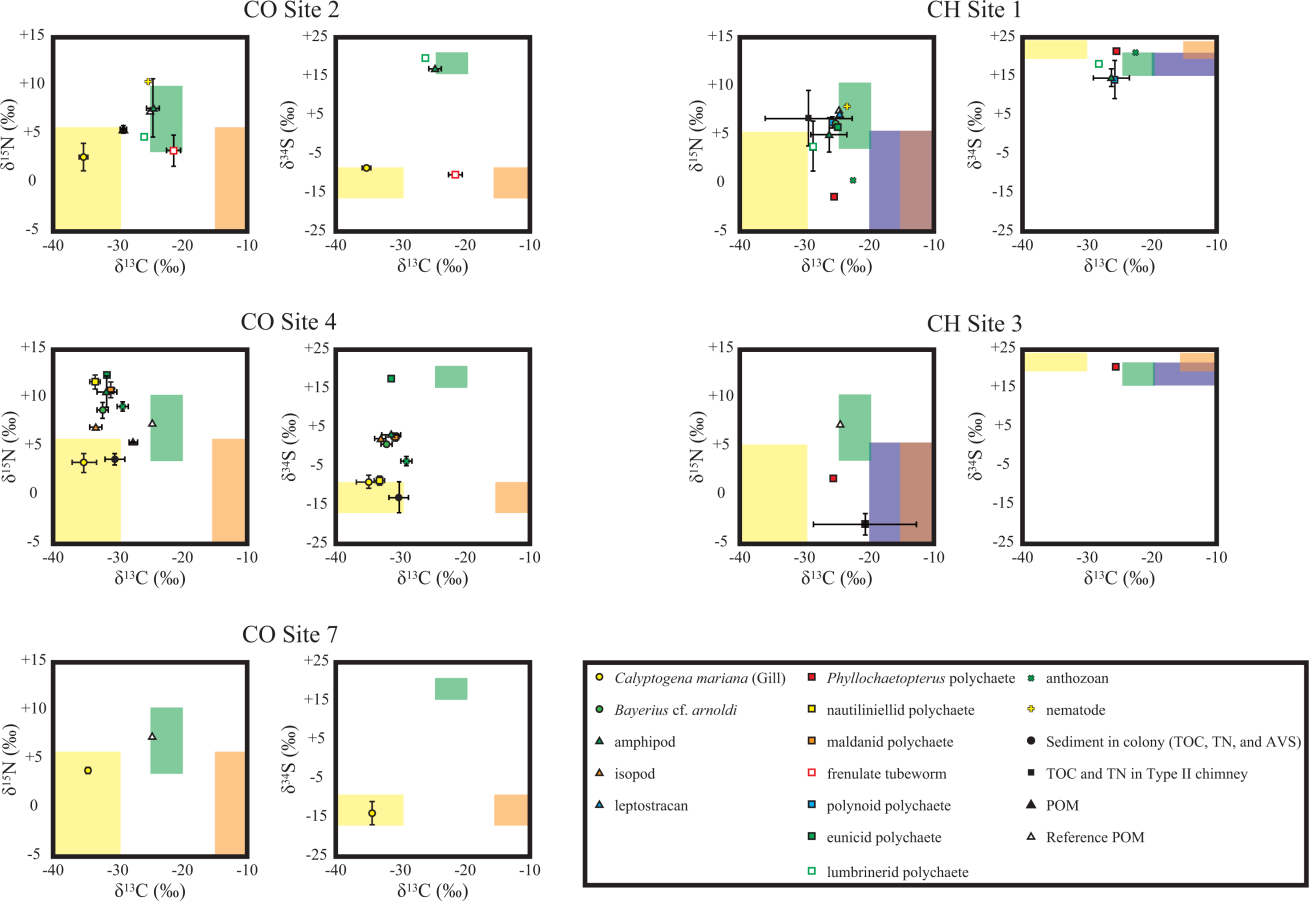
Plots of carbon versus nitrogen and carbon versus sulfur isotopic compositions of animal soft tissues obtained from CO (a, b, and c) and CH (d and e) Sites in the SSF. Yellow, orange, blue, and green shadows represent the expected ranges of the isotopic compositions of thiotroph-CBB, thiotroph-rTCA, methanotroph, and photosynthetically-derived organic matter, respectively. In CO Sites, the ranges of isotopic signatures of methanotroph were not shown due to the low δ^13^C value (*c*. −48‰).

The nautiliniellid polychaetes inhabiting inside the clam shells had δ^13^C of −33.4 ± 0.8‰ and δ^34^S of −9.0 ± 1.1‰ that were comparable to those of the host clams (−33.5 ± 1.4‰ and −7.4 ± 2.6‰, respectively) (t-test, *p* > 0.05 for δ^13^C and δ^34^S values). In turn, the nautiliniellid polychaetes had ~4‰ higher δ^15^N values (+11.7 ± 0.7‰) than those of the host clam tissues (t-test, *p* < 0.05) (Table 8 and Figs 4 and 5).

The amphipods and lumbrinerid polychaetes in CO Site 2, and the eunicid polychaetes in CO Site 4 had δ^13^C values of −24.6 ± 1.0‰, −26.1‰, and −31.7‰, respectively (Table 8). These animals had high δ^34^S values (+16.6 ± 1.5‰, +19.8‰, and +17.2‰), that close to those of seawater sulfate (+21‰ [46]). The δ^15^N values of the lumbrinerid polychaetes (+4.9‰) were clearly lower than that of the reference POM (+7.5‰). While the amphipods had variable δ^15^N values (+7.7 ± 3.0‰), the eunicid polychaetes had the highest δ^15^N value of +12.4‰ in the SSF.

The isopods, *B*. cf. *arnoldi*, maldanid polychaetes, and amphipods in CO Site 4 had similar δ^13^C and δ^34^S values (one-way ANOVA, *p* > 0.05) of *c*. −32‰ and *c*. +2‰, respectively (Table 8). The δ^13^C and δ^34^S values of these animals are lower than those of photosynthetically-derived organic matter and seawater sulfate, respectively (Fig 4). These animals had significantly different δ^15^N values (one-way ANOVA, *p* < 0.05) from +7.1 ± 0.1‰ (isopods) to +10.8 ± 0.1‰ (maldanid polychaetes).

The anthozoans were also characterized by a relatively low δ^13^C (−29.2 ± 0.9‰) and δ^34^S (−3.9 ± 1.2‰) values. The anthozoans had relatively high δ^15^N value of +9.2 ± 0.5‰. The nematode from CO Site 2 had a δ^13^C value (−25.3‰) comparable to the photosynthetic signature and the highest δ^15^N value (+10.4‰) among the animals in the CO Sites.

### Animals in CH Sites

The δ^13^C and δ^34^S values of all the animals in CH Sites (*Phyllochaetopterus* polychaetes, anthozoans, amphipods, lumbrinerid polychaetes, polynoid polychaetes, eunicid polychaetes, leptostracans, *B*. cf. *arnoldi*, and nematodes) ranged between −28.4 and −22.3‰ and between +14.3 and +21.6‰, respectively (Fig 4). The δ^13^C and δ^34^S values of the CH Sites animals are comparable to those of the photosynthetic organic matter and seawater sulfate, respectively. In addition, the δ^13^C and δ^34^S values of the CH Sites animals are generally higher than those of the CO Sites animals (Fig 4). Moreover, most of the δ^15^N values of animals in CH Sites were lower than that of the reference POM (+7.5‰) while the nematodes had δ^15^N values of +8.0‰ (Fig 4).

## Discussion

### Evidence for active shimmering of serpentinization-associated fluid from the SSF

The highly alkaline fluids occupying the GATS-blue (pH = 9.89) and chimney pore waters (pH = 10.0) demonstrate serpentinization-involved geofluid actively emits from the SSF seafloor. The pH value of the fluids are in fact as high as those observed in other serpentinization-associated seafloor geofluid systems (9–9.8 for Lost City hydrothermal field [12]; 12.5 for South Chamorro Seamount [55]). The highly alkaline fluids rich in reducing chemical species, such as methane and hydrogen sulfide, are confirmed and can provide available energy and nutrient sources for chemosynthetic community. It is noted that the distinct chemical compositions between the two GATS fluid samples, especially in pH, suggest that the original fluid chemistry of fluids emitting around each GATS may be different. However, it remains unclear whether the differences reflect the original fluid chemistry or not due to a possible alteration of fluid chemistry within the GATS chamber until the sampling. A consistency of fluid chemistry between GATS-blue and chimney porewaters (Fig 3) nevertheless implies appropriate sampling at least for the GATS-blue.

The δ^34^S values of sulfate in the GATS fluids, comparable to the ambient seawater sulfate-sulfur, suggest that sulfate in the GATS fluids was derived from the ambient seawater. The sulfate concentrations of the GATS fluids are thus hypothesized as an indicator for a mixing between two end-member fluids, the sulfate-rich seawater ([SO_4_^2−^] = 28 mM) and the sulfate-free seeping fluid ([SO_4_^2−^] = 0 mM). The mixing model based on the sulfate concentration (Fig 3) can provide estimated fluid chemistry of the seeping end-member. The estimated “negative” Mg^2+^ and Si concentrations in the SSF seeping fluid (Fig 3) are consistent with those obtained from the Lost City hydrothermal vent fluids [12] and pore water fluids of the Mariana serpentinite mud volcanoes [55] as well as the trend of experimental study in the serpentinization of peridotite [56]. They can be explained by Mg precipitation from a mixed fluid between the Mg-enriched seawater and the Mg-free seeping fluid. The Mg depletion in the seeping fluid seems reasonable because water/rock interaction during the exothermic serpentinization removes Mg from the liquid phase [12] while the precipitation of brucite from the mixed fluid due to high pH condition further removes seawater-derived Mg. The precipitation of Mg minerals from the mixing fluid is suggested by the brucite-like whitish precipitation observed in the GATS-blue marker.

The ^34^S-enriched H_2_S in the GATS fluid can be produced from more ^34^S-enriched sulfate through microbial sulfate reduction (MSR) activity [23]. As ^32^S-preferential sulfate consumption by subseafloor SRB populations and subsequent subseafloor deposition of sulfide minerals such as pyrite remove ^32^S from the ascending fluid, and the remnant ^34^S-enriched sulfate at the later stage of MSR occurring on the seafloor could be a source of ^34^S-enriched H_2_S in the GATS fluid. The ^34^S-enriched H_2_S may also reflect complex sulfur cycles carried out by the microbial population [57] in the GATS marker during approximately one year until sampling. However, the GATS-blue fluid contained very high levels of H_2_S relative to venting fluid at the Lost City (<64 μM [12]), while pore water fluids of serpentinite mud volcanoes contain a similar level of H_2_S (0.2–1.3 mM [55]). Although sulfate are plausibly lacking in fluids from the SSF and the Lost City field [12], the sulfate concentrations in pore fluids of the serpentinite mud volcanoes are as high as ambient seawater [55]. Those results suggest that the subsurface microbial activity, especially MSR, is sufficiently active to deprive the SSF fluid of sulfate.

Major ion concentrations of the estimated SSF end-member fluid, comparable to those of the seawater, suggest that water source of the SSF fluid may be seawater penetrating the crust around the SSF rather than the slab-derived fluid. If dehydration of the subducting slab occupies a majority of the fluid source discharging at the SSF, like in forearc serpentinite seamounts, major ion concentrations of the estimated end-member fluid would be different from those of the seawater due to deep subseafloor processes through geological time spans [58]. However, such trends could not be found in the estimated SSF fluids chemistry (see Fig 2). In turn, fresh basaltic glass associated with igneous activity of the backarc basin was recognized near the SSF area [59], and the igneous activity might be as a possible driving force for fluid circulation of the SSF geofluid system like seafloor hydrothermal system.

### Chemosynthetic primary production in the sediments beneath clam colonies and in the chimneys in the SSF

In the SSF, chemosynthetic primary production in the sediments beneath the clam colony and in the chimneys likely accounts for the main food sources for the animals as the great biomass of local benthic community cannot be sustained only by phytoplankton-derived sinking organic matter. The large variation in δ^13^C values of sedimentary TOC beneath the clam colony suggests that there are more than two primary productions such as thiotroph-CBB, thiotroph-rTCA, and/or methanotrophs. These chemosynthetic activities are plausibly supported not only by methane and sulfide derived from deep-sourced ascending fluid but also by biogenic sulfide through MSR within sediments. The MSR in the sediment is fueled by the ascending fluid, because the AVS levels increases with depth. Therefore, the fluid is expected to contain some electron donors for SRB such as organic compounds, methane and/or hydrogen (e.g. [6, 23]).

The chimneys are thought to form by precipitation minerals originating in the ascending fluid associated with serpentinization of mantle peridotite and have been classified into three types [10] as mentioned above: (1) the Type I brucite-rich chimney has microbial mats but a lack of colonized animal under higher fluid flux condition of the highly alkaline and reduced fluid, (2) the Type II carbonate-rich chimney has obvious microbial mats and densely-colonized animals on it under lower fluid flux condition, (3) the Type III brucite-poor chimney is dissolving and alternating after the fluid discharge has ceased.

The Type I chimney had low TOC and TN levels in the inner part of the chimney that is consistent with the visual observation of low biological accumulation [10]. As marine microbial organic matter generally has a TOC/TN atomic ratio of 4 to 6 [60, 61], the high TOC/TN atomic ratios together with low TOC and TN levels of the Type I chimney suggest the occurrence of *in situ* organic matter decomposition that increases TOC/TN atomic ratio. The high δ^15^N value (up to +11.4‰) of the inner part of the chimney is not conflict with this interpretation [62]. Thus, chemosynthetic primary production is considered to be active on the surface of Type I chimneys and all sections of porous Type II chimneys, which have low δ^15^N values and TOC/TN atomic ratios.

The variation of δ^13^C values of the TOC on the surface of Type I chimneys and all sections of porous Type II chimneys possibly reflect the compositional and functional diversity of microbes in the fluid and/or chimney structures (Table 5). For example, thiotroph-CBB, which has a low δ^13^C value (−35‰), could be dominant in the microbial community in the surface of lower and middle sections of the chimneys (Table 5). On the other hand, thiotroph-rTCA and/or methanotrophs, which have a high δ^13^C value (−15‰ and −22 to −9‰, respectively), were expected to be dominant in the root and upper sections of the chimney surfaces. Another possible explanation for this wide range of the δ^13^C values of the TOC may be due to differences in carbon source availability from seeping fluids, as observed in the Lost City hydrothermal field, where remarkable ^13^C-enriched organic matters were reported from carbonate chimneys and fissures associated with an extremely carbon-limited environment (−27.7 to −2.8‰; [13, 63, 64]). The wide range of δ^13^C values in the carbonate may support the notion that the differences of dissolved inorganic carbon (DIC) availability or the δ^13^C of the source DIC affect the isotopic signature of producers. Further studies, such as microbial community analyses, biomarker compositions and compound-specific isotope analysis, may reveal the sources of the organic matter in more detail.

On the other hand, on the surface of the largely lithified Type II chimney sample (Chim 2, Table 5), relatively high δ^15^N values and low TOC/TN atomic ratios suggest that organic matter with high TOC and TN levels may reflect incorporation of photosynthetic organic matter as the ultimate source. This suggests that the remains of organisms feeding on suspended organic matter are incorporated in the chimney surface. The δ^13^C values of carbonates in the chimney surfaces were similar to that of DIC in ambient seawater (*c*. 0‰), indicating that the source of DIC forming surfaces of the carbonate chimney was provided mainly from ambient seawater. This suggests that the supply of reduced chemical species from the ascending fluid is limited to the lithified Type II chimney surface.

### Evaluation of basal foods and food-web structure of the animal community

#### Clam colony sites

The *Calyptogena* clams and the frenulate tubeworms are expected to completely rely on symbionts for their energy and nutrients. Their symbionts are typically occupied by thiotrophic microbes (e.g. [7, 65, 66]), and the δ^34^S values of these animals are indeed identical to those of AVS (−10‰) in surface sediments. These biological and isotopic characteristics strongly suggest that the proportions of methanotrophic and photosynthetic products to their nutrient sources are negligible (*f*_mt_ = *f*_pp_ ≈ 0). It allows their basal carbon sources calculated from the δ^13^C values based on Eq 1. The calculation yields that CBB-derived carbon occupies nearly 100% for the clams and approximately half (40%) for the tubeworms (Table 9 and Fig 5).

**Table 9:** Calculation results of proportions of each basal source for animals in CO Sites.

**Fauna**	Site	Thiotroph-CBB (*f*_tc_)	Thiotroph-rTCA (*f*_tr_)	Methanotroph (*f*_mt_)	Photosynthetic production (*f*_pp_)	n
		**(%)**	**(%)**	**(%)**	**(%)**	
*Calyptogena mariana*	CO Site 2	101 ± 3	-1 ± 3	-	-	3
	CO Site 4	101 ± 8	-1 ± 8	-	-	3
	CO Site 7	98 ± 2	2 ± 2	-	-	3
frenulate tubeworm	CO Site 2	40 ± 5	60 ± 5	-	-	2
nautiliniellid polychaete	CO Site 4	94 ± 3	4 ± 3	-	-	8
lumbrinerid polychaete	CO Site 2	-	-	6	94	1
amphipod	CO Site 2	-	-	0 ± 4	100 ± 4	3
	CO Site 4	57	43	3		
eunicid polychaete	CO Site 4	-	-	30	70	1
isopod	CO Site 4	60	40	3		
*Bayerius* cf. *arnoldi*	CO Site 4	65 ± 1	35 ± 1	2		
maldanid polychaete	CO Site 4	59 ± 3	41 ± 3	3		
anthozoan	CO Site 4	80 ± 4	20 ± 4	4		
nematode	CO Site 2	unknown	unknown	unknown	unknown	1

-; assumed 0%

As majority of the organic matter in *Calyptogena* gill is typically occupied by endosymbiont’s cells [54], δ^15^N values of the clam gill (*c*. +3‰; Table 8) can be regarded as a representative value of the primary producer for the ^15^N-based trophic level estimation. The higher δ^15^N values of the other tissues than those of gill are reasonable as a higher trophic level from the symbiont. In addition, as the δ^15^N value of the thioautotrophs estimated here (*c*. +3‰) is lower than that of the photosynthetic product (+7.5‰), the δ^15^N value of animals not higher than *c*. +7.5‰ is a likely indication for significant contribution of the chemosynthetic products in the CO Sites.

The nautiliniellid polychaetes, sampled from the mantle cavities of the *Calyptogena* clam, are expected to obtain energy and nutrients from the host clam. Indeed, the carbon and sulfur isotopic compositions of the nautiliniellid polychaetes suggest the thiotroph-CBB as a majority carbon and sulfur sources (95%) as well as the host clam (Table 9 and Fig 5). The δ^15^N values (*c*. +12‰) of the nautiliniellid polychaetes, which is *c*. 4‰ higher than that of the clam body (*c*. ≤+8‰) and corresponds to an increase of the trophic level, are consistently explained by the life style.

The amphipods and the lumbrinerid polychaetes in CO Site 2 had the δ^34^S values as high as *c*. +20‰ that indicate little contributions of thiotrophic products for their basal sulfur sources (*f*_tc_ = *f*_tr_ ≈ 0) based on Eq. 4. The δ^13^C values and Eq. 1 with an assumption of both *f*_tc_ and *f*_tr_ of 0 yield that the photosynthetic-derived carbon likely occupies nearly 100% for their basal carbon sources (Table 9 and Fig 5). The δ^15^N values of these animals were highly diverse even within the CO Site 2 that did not allow us to evaluate their trophic level. Although possible δ^15^N heterogeneity within an individual [67] may cause the variation, reasons remain unidentified at this moment.

The eunicid polychaetes in CO Site 4 also had the high δ^34^S values (>+15‰) that suggest slight contribution of thiotrophic products as food sources (*f*_tc_ = *f*_tr_ ≈ 0). Their δ^13^C values and Eq. 1 yield that the eunicid polychaetes rely a large part (*c*. 70%) of the basal carbon source on the photosynthesis-derived organic matter. The high δ^15^N value (+12.4‰) is consistent with the digestion of the photosynthesis-derived organic matter. However, its potential preys could not be found among the animals in the present analyses (Fig 4). This suggests that other animals in the site that do not rely on thioautotrophic products and are preyed upon by the eunicid polychaetes, but we did not sample them.

The isopods, the snail *B*. cf. *arnoldi*, the maldanid polychaetes, and the amphipods in CO Site 4 had the intermediate δ^34^S values between the δ^34^S_sulfide_ and δ^34^S_sulfate_ (Fig 4) that prevents us from estimating the proportions among each four basal source. In turn, the intermediate δ^34^S values and Eq. 4 yield that approximately half of their basal food sources are likely derived from the chemosynthetic (thiotrophic) products (Table 9). The low δ^15^N values of isopods (*c*. +7‰), comparable to those of the syntrophic *Calyptogena* body, imply that the isopods directly uptake chemosynthetic microbes. The high δ^15^N values (*>*+9‰) of the snails, maldanid polychaetes, and the amphipods imply that trophic levels of these animals were likely higher than those of the isopods. This isotopic suggestion seems consistent with behaviors of *B*. cf. *arnoldi* who behaves as carnivore in various communities [68]. Both δ^13^C and δ^34^S values are similar between the snails and isopods (Fig 5), implying their similar basal foods. It is also the case for the maldanid polychaetes (Fig 5) while the maldanid polychaetes in marine sediments behave deposits feeder or detritus feeders [69].

The anthozoans, known as suspension feeder, in the CO Site 4 had ~3‰ higher δ^15^N values than the suspended POM around their habitat (Figs 4 and 5), supporting the suspended POM feeding. It is also supported by similar δ^13^C values between the anthozoans and POM. In turn, the δ^34^S values (*c*. −4‰) of anthozoans indicate that majority of their nutrient sources were derived from the thiotrophic microbes (*f*_tc_ + *f*_tr_ ≈ 0.8). Because the suspended POM had the intermediated δ^13^C and δ^15^N values between those of sediment beneath the clam colony and the photosynthetic product (δ^34^S was not obtained), the thiotrophy-derived sulfur would be supplied by re-suspension of the sediment. The basal food source of the nematodes in CO Site 2 cannot be constrained due to lack of δ^34^S measurement, the intermediate δ^13^C value among the sources, and the δ^15^N value higher than +9‰.

### Chimney sites

The δ^15^N values of the animals in the CH Sites that were lower than +7.5 strongly suggest significant contributions of chemosynthetic-derived nitrogen as the nitrogen nutrient source. It is however difficult to strictly constrain proportions of each chemosynthetic metabolism due to the δ^13^C value of methane (−21.5 to −8.6‰) comparable to those of photosynthesis (−25‰) and thiotroph-rTCA (−12.5‰) as well as the δ^34^S_sulfide_ value (+24.2‰) comparable to δ^34^S_sulfate_ (+24‰) (Table 4).

The *Phyllochaetopterus* polychaetes and the anthozoans, colonized on/in the chimneys and attached to chimney walls, respectively, had the lowest δ^15^N values (*c*. +0‰) in the CH Sites animals (Fig 4 and Table 8). As the *Phyllochaetopterus* polychaetes and the anthozoans are known to suspension feeders [70], they likely relied on the chemosynthetic products attached on the chimney and from the suspended POM. The δ^15^N values of the other animals in CH Sites are ≥3.5‰ higher than those of the *Phyllochaetopterus* polychaetes and the anthozoans, suggesting that the others were secondary (or higher) consumers. The lumbrinerid and the polynoid polychaetes had the δ^13^C values lower than the reference POM. It suggests a certain amount of thiotroph-CBB-derived carbon contributing to their basal food source although actual foods digested remain unknown.

## Conclusion

In this study, we collected fluid, sediment, chimney, and animal samples from the deepest known serpentinite-hosted animal communities named SSF. Seeping fluids recovered by trapping in the GATS markers had distinct chemical compositions from the ambient seawater. Particularly, high pH of the accumulated GATS fluid is the first direct evidence for on-going seepage of serpentinization-associated geofluid in this area. The highly alkaline fluids rich in hydrogen sulfide and methane can serve as an energy and nutrient sources to sustain chemosynthetic productions and associated animal communities. Carbon, nitrogen, and sulfur isotope systematics of various components in the SSF suggest that most of the animals living there rely on chemosynthetic production. On the other hand, some animals use not only chemosynthetic products but also photosynthetic products. It means that sinking particles from the euphotic zone are also important nutritional sources for animals inhabiting the SSF, despite being at a depth of ~5,700 m.
